# *cis*-Decoder discovers constellations of conserved DNA sequences shared among tissue-specific enhancers

**DOI:** 10.1186/gb-2007-8-5-r75

**Published:** 2007-05-09

**Authors:** Thomas Brody, Wayne Rasband, Kevin Baler, Alexander Kuzin, Mukta Kundu, Ward F Odenwald

**Affiliations:** 1 Neural Cell-Fate Determinants Section, NINDS, NIH, Bethesda, MD, 20892, USA; 2 Office of Scientific Director, IRP, NIMH, NIH, Bethesda, MD, 20892, USA

## Abstract

A systematic approach is described for analysis of evolutionarily conserved *cis*-regulatory DNA using *cis-*Decoder, a tool for discovery of conserved sequence elements that are shared between similarly regulated enhancers. Analysis of 2,086 conserved sequence blocks (CSBs), identified from 135 characterized enhancers, reveals most CSBs consist of shorter overlapping/adjacent elements that are either enhancer type-specific or common to enhancers with divergent regulatory behaviors. Our findings suggest that enhancers employ overlapping repertoires of highly conserved core elements.

## Abstract 2

: The use of *cis*-Decoder, a new tool for discovery of conserved sequence elements that are shared between similarly regulating enhancers, suggests that enhancers use overlapping repertoires of highly conserved core elements.

## Background

Tissue-specific coordinate gene expression requires multiple inputs that involve dynamic interactions between sequence specific DNA-binding transcription factors and their target DNAs. The enhancer or *cis*-regulatory module is the focal point of integration for many of these regulatory events. Enhancers, which usually span 0.5 to 1.0 kb, contain clusters of transcription factor DNA-binding sites (reviewed by [1-3]). DNA sequence comparisons of different co-regulating enhancers suggest that many may rely on different combinations of transcription factors to achieve coordinate gene regulation. For example, the *Drosophila *pan-neural genes *deadpan*, *scratch *and *snail *all have distinct central nervous system (CNS) enhancers that drive expression in the same embryonic neuroblasts, yet comparisons of these enhancers reveal that they have few sequences in common [4,5].

Comparative genomic analysis of orthologous *cis*-regulatory regions reveals that many contain multi-species conserved sequences (MCSs; reviewed by [6-8]). Close inspection of enhancer MCSs reveals that these sequences are made up of smaller blocks of conserved sequences, designated here as 'conserved sequence blocks' (CSBs). *EvoPrint *analysis of enhancer CSBs reveals that many have remained unchanged for over 160 million years (My) of collective divergence [9] (and see below). CSBs that are over 10 base-pairs (bp) long are likely to be made up of adjacent or overlapping sequence-specific transcription factor DNA-binding sites. For example, DNA-binding sites for transcription factors that play essential roles in the regulation of the previously characterized *Drosophila Krüppel *central domain enhancer [10-12] are found adjacent to or overlapping one another within enhancer CSBs [9]. Although transcription factor consensus DNA-binding sites are detected within CSBs, searches of 2,086 CSBs (27,996 total bp) curated from 35 mammalian and 99 *Drosophila *characterized enhancers reveal that well over half of the sequences do not correspond to known DNA-binding sites and, as yet, have no assigned function(s) (this paper).

**Figure 1:**
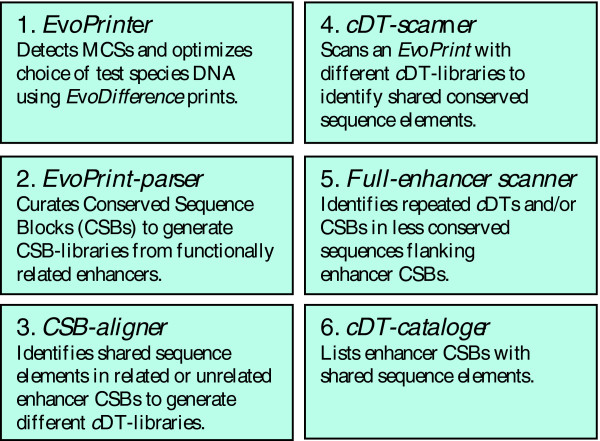
*cis*-Decoder methodology for identification of conserved sequence elements shared among different enhancers. The *cis*-Decoder methodology allows one to discover short 6 to 14 bp sequence elements within conserved enhancer sequences that are shared by other functionally related enhancers or are common to many enhancers with divergent regulatory behaviors. These shared sequence elements or *c*DTs can be used to identify and differentiate between *cis*-regulatory enhancer regions that regulate different tissue-specific expression patterns. *cis*-Decoder analysis involves the sequential use of the following web-accessed computer algorithms: *EvoPrinter *→ *EvoPrint-parser *→ *CSB-aligner *→ *cDT-scanner *→ *Full-enhancer scanner *→ *cDT-cataloger*.

In order to initiate the functional dissection of novel CSBs and to gain a better understanding of their substructure, we have developed a multi-step protocol and accompanying computer algorithms (collectively known as *cis*-Decoder; see Figure 1) that allow for the rapid identification of short 6 to 14 bp DNA sequence elements, called *cis*-Decoder tags (*c*DTs), within enhancer CSBs that are also present in CSBs from other enhancers with either related or divergent functions. There is no limit to the number of enhancer CSBs examined by this approach, which allows one to build large *cDT-libr*aries. Due to their different copy numbers, positions and/or orientations within the different enhancers, the conserved short sequence elements may otherwise go unnoticed by more conventional DNA alignment programs. Because this approach does not rely on any previously described transcription factor consensus DNA-binding site information or any other predicted motif or the presence of overrepresented sequences, *cis*-Decoder analysis affords an unbiased 'evo-centric' view of shared single or multiple sequence homologies between different enhancers. The *cDT-libr*aries and *cis-*Decoder alignment tools enable one to differentiate between functionally different enhancers before any experimental expression data have been collected. *cis*-Decoder analysis reveals that most CSBs have a modular structure made up of two classes of interlocking sequence elements: those that are conserved only in other enhancers that regulate overlapping expression patterns; and more common conserved sequence elements that are part of divergently regulated enhancers.

To demonstrate the efficacy of *cis*-Decoder analysis in identifying shared enhancer sequence elements, we show how *c*DT-library scans of different *EvoPrinted *mammalian and *Drosophila *enhancers accurately identify shared sequences within enhancers involved in similar regulatory behaviors. The *cis*-regulatory regions of the mammalian Delta-like 1 (Dll1) and *Drosophila snail *genes, which contain closely associated neural and mesodermal enhancers, were selected to highlight *cis*-Decoder's ability to differentiate between enhancers with different regulatory functions. We show how a *c*DT-library generated from both mammalian and *Drosophila *enhancer CSBs can be used to identify enhancer type-specific elements that have been conserved during the evolutionary diversification of metazoans. Finally, we show how *cis*-Decoder analysis can be used to examine novel putative enhancer regions.

## Results and discussion

### Generation of *EvoPrints *and CSB-libraries

Our analysis of mammalian *cis*-regulatory sequences included 14 neural and 21 mesodermal enhancers whose regulatory behaviors have been characterized in developing mouse embryos. A full list of enhancers used in this study and the references describing their embryonic expression patterns is given in Table 1. In most cases, their *EvoPrints *included orthologs from placental mammals (human, chimp, rhesus monkey, cow, dog, mouse, rat) or also included the opossum; these species afford enough additive divergence (≥200 My) to resolve most enhancer MCSs [13]. When possible, chicken and frog orthologs were also included in the *EvoPrints*. Except when *EvoDifference *profiles [9] revealed sequencing gaps or genomic rearrangements in one or more species that were not present in the majority of the different orthologous DNAs, pair-wise reference species versus test species readouts from all of the above BLAT formatted genomes [14] were used to generate the *EvoPrints*.

**Table 1:** Enhancers analyzed

Enhancer	Class	Reference
***Drosophila***		
*anterior open/yan*	neur	[61]
*atonal *F:2.6 PNS	neur	[62]
*bagpipe *DS3.5	meso	[63]
*bearded *PNS	neur	[57]
*biparous/tap *CNS	neur	[64]
*charlatan *PNS	neur	[65]
*deadpan *CNS	neur	[5]
*deadpan *PNS	neur	[5]
*dpp *813	meso	[28]
*eve *neuronal CNS	neur	[66]
*eve *EL CNS	neur	[18]
*eve *MES	meso	[67]
*eve *stripe 1	seg	[18]
*eve *stripe 2	seg	[68]
*eve *stripe 4+6	seg	[18]
*eve *stripe 5	seg	[18]
*eve *stripe 3+7	seg	[69]
*eve ftz*-like	seg	[18]
*eyeless *12 PNS	neur	[16]
*ftz *distal	meso	[70]
*ftz *proxA	meso	[70]
*ftz *CE8024	seg	[71]
*ftz *neuro CNS	neur	[72]
*ftz *PS4*	seg	[70]
*giant *1	seg	[24]
*giant *3	seg	[24]
*giant *6	seg	[24]
*giant *10	seg	[24]
*gooseberry-n *CNS	neur	[73]
*gooseberry *GLE	neur	[74]
*gooseberry *fragIV	seg	[74]
*hairy *h7	seg	[75]
*hairy *stripe 0	seg	[44]
*hairy *stripe 1	seg	[17]
*hairy *stripe 5	seg	[76]
*hairy *stripe 3+4	seg	[77]
*hairy *stripe 6+2	seg	[77]
*heartless *early	meso	[78]
*huckebein *ventral	seg	[79]
*hunchback *CNS	neur	[19]
*hunchback *ant	seg	[80]
*hunchback *upstr	seg	[20]
*knirps *5	seg	[24]
*Krüppel *CD1	seg	[10]
*mir-1*	meso	[81]
*Mef2 *I-D	meso	[82]
*Mef2 *II-E	meso	[79]
*nerfin-1 *CNS	neur	AK (pers. com.)
*odd skipped*-3	seg	[24]
*odd skipped*-5	seg	[24]
*paired *cc	seg	[80]
*paired *O-E	seg	[44]
*paired *stripe P	seg	[83]
*paired *stripe 1	seg	[84]
*paired *stripe 2P	seg	[83]
*paired *zebra	seg	[79]
*pdm-1 *Gap+CNS	seg/n	[84]
*pdm-2 *CE8012	neur	[71]
*pdp1 *intron 1	meso	[85]
*pdp1 *intron 2	meso	[85]
*runt *stripe 1E+6	seg	[86]
*runt *stripe 1+7	seg	[86]
*runt *stripe 3+7	seg	[86]
*runt *stripe 5	seg	[86]
*runt *15G CNS	neur	[86]
*Schizo/loner *PNS	neur	[65]
*scratch *sA	neur	[5]
*scratch *PNS	neur	[5]
*Scr *3.0RR	meso	[23]
*Scr *7.0RR	meso	[23]
*Scr *8.2XX	meso	[23]
*scute *SCM	neur	[87]
*serpent*-A7.1EB	meso	[22]
*snail *CNS	neur	[4]
*snail *PNS	neur	[4]
*snail *MES	meso	[4]
*string *b-5.8 CNS	neur	[88]
*teashirt *del-1-5	meso	[89]
*tinman *B	meso	[21]
*tinman *C	meso	[21]
*tinman *D	meso	[21]
*toll*-6.5RL	meso	[90]
*β-tub *56DAS1	meso	[91]
*Tropomyosin1*-M	meso	[92]
*Tropomyosin1*-P	meso	[92]
*twist*-del	meso	[48]
*vnd *CNS	neur	[93]
*vnd *A CNS	neur	[93]
*wor *CNS	neur	[94]
		
**Mammalian**		
*bagpipe *Hox 1	meso	[95]
Cbfa1 non-coding	meso	[96]
Dll1 HI CNS	neur	[35]
Dll1 HII CNS	neur	[35]
Dll1 msd	meso	[35]
Dll1 msd II	meso	[35]
*forkhead *box f1	meso	[97]
Gata6	meso	[38]
dHAND	meso	[98]
Hes 7	meso	[99]
HoxA-5	meso	[100]
H. domain only	neur	[101]
IA-1 CNS	neur	[102]
α7 integrin	meso	[103]
Mef2c	meso	[104]
Mash1 CNS	neur	[105]
Math1 CNS	neur	[27]
Myogenic factor-5	meso	[106]
Nestin CNS	neur	[107]
Nfatc1	meso	[108]
Neurogenin 2:5'	neur	[109]
Neurogenin 2:3'	neur	[109]
Nkx-2.5	meso	[110]
Otx 2 CNS	neur	[111]
Pax 3	meso	[112]
Phox2b CNS	neur	[25]
Serum response f	meso	[113]
Six2	meso	[114]
Sox-2 CNS	neur	[115]
Sox-2 #2 CNS	neur	[116]
Sox 9^p ^CNS	neur	[37]
Stem cell leukemia	meso	[117]
Tbx1	meso	[118]
Wnt-1	neur	[36]

Meso, mesodermal; neur, neural; seg, segmental.

Using the *EvoPrint*-*Parser *program, both forward and reverse-complement sequences of each enhancer CSB of 6 bp or greater were extracted, named and consecutively numbered. Based on their enhancer regulatory expression pattern, CSBs were grouped into two different CSB-libraries, neural and mesodermal (Tables 1 and 2). Although there exists a distinction between expression in either neural or mesodermal tissues, each of the CSB-libraries represent a heterogeneous population of enhancers that drive gene expression in different cells and/or different developmental times in these tissues. For this study, CSBs of 5 bp or less were not included in the analysis. Although these shorter CSBs, particularly the 5 and 4 bp CSBs, are most likely important for enhancer function, the use of CSBs of 6 bp or larger (representing greater than 80% of the conserved MCS sequences) is sufficient to resolve sequence element differences between enhancers that regulate divergent expression patterns (see below). A total of 286 neural CSBs and 289 mesodermal CSBs were extracted from the mammalian enhancers (Table 2).

**Table 2:** *cis*-Decoder libraries

*cis*-Decoder tag libraries	*c*DTs	Enhancers	CSBs/Total bp
**Mammalian/vertebrate**			
Neural specific	336	14	286/4,162
Mesodermal specific	258	21	289/3,749
Common	137	35	575/7,911
Neural enriched*	60	35	575/7,911
Mesodermal enriched*	55	35	575/7,911
			
***Drosophila***			
Neural specific	444	36	601/8,002
Segmental specific	284	38	513/6,608
Mesodermal specific	169	25	398/5,469
Neural and segmental	451	75	1,114/14,610
Neural/segmental enriched*	277	100	1,511/20,085
Mesodermal enriched*	104	63	1,511/20,085
Common	993	100	1,511/20,085
			
***Drosophila*/mammalian/vertebrate**			
Neural specific	873	50	887/12,164
Mesodermal specific	445	46	687/9,218

* cDTs have a ≥75% correspondence to a specific library but are also present at a low frequency in unrelated enhancers.

For *Drosophila*, three CSB-libraries, neural, segmental and mesodermal, were generated from CSBs identified by *EvoPrinting *(Tables 1 and 2): neural enhancers included those regulating both CNS and peripheral nervous system (PNS) determinants; segmental enhancers included those regulating both pair-rule and gap gene expression; and mesodermal enhancers included those regulating both presumptive and late expression. Many of the *D. melanogaster *reference sequences used to initiate the *EvoPrints *were curated from the regulatory element database *REDfly *[15], while others were identified from their primary reference (Table 1). The collection of neural enhancers includes both those that direct expression during early development, such as the *snail *[4], *scratch*, and *deadpan *CNS and PNS enhancers [5], and late nervous system regulators, such as the *eyeless *enhancer *ey*12 [16], which confers expression in the adult brain. The early embryonic segmental enhancers represent pair-rule regulators such as the *hairy *stripe 1 [17] and *even-skipped *stripe 1 [18] enhancers, and gap expression regulators, such as the *hunchback *enhancers [19,20]. The mesodermal enhancers include those directing mesodermal anlage expression of *snail *[4] and *tinman *[21], and late expressing enhancers, such as those directing *serpent *fat body expression [22] and mesodermal expression of *Sex combs reduced *[23]. The collective evolutionary divergence of all of the *EvoPrints *was greater than 100 My and in most cases *EvoPrints *represented over approximately 160 My of additive divergence. The average CSB length for both the *Drosophila *and mammalian CSBs is 13 bp; the longest identified CSBs were 99 bp from the *giant (-10) *segmental enhancer [15,24] and 95 bp from the Paired-like homeobox-2b mammalian neural enhancer [25]. Complete lists of all CSBs identified in this study are given at the *cis-*Decoder website [26].

### Identification and use of *cis*-Decoder tags

As an initial step toward understanding the nature of the CSB substructure, we have developed a set of DNA sequence alignment tools, known collectively as *cis*-Decoder, that allow identification of 6 bp or greater perfect match identities, called *c*DTs, within two or more CSBs from either similar or divergent enhancers. The *c*DTs, which range in size from 6 to 14 bp with an average of 7 or 8 bp, are organized into *c*DT-libraries that identify sequence elements within CSBs of the same CSB-library. In addition, common *c*DT-libraries that represent sequence elements aligning to CSBs of two or more different CSB-libraries were also organized.

Mammalian CSB alignments, using the *CSB-aligner *program, yielded 336 neural specific and 60 neural-enriched *c*DTs and analysis of the mammalian mesodermal CSBs yielded 258 mesodermal specific and 55 mesodermal enriched *c*DTs (Table 2). The CSB alignments also produced 137 *c*DTs that are common to both neural and mesodermal CSBs. Alignments of the *Drosophila *enhancer CSBs yielded 444 neural specific *c*DTs (showing no hits on mesodermal or segmental enhancer CSBs), 284 segmental enhancer specific *c*DTs and an additional 451 *c*DTs found in neural and segmental enhancers but not part of mesodermal CSBs (Table 2). We also identified 451 *cDTs *that were enriched in neural and/or segmental CSBs but were also found at a lower frequency in mesodermal enhancer CSBs. From the mesodermal CSBs analyzed, 169 mesodermal specific *c*DTs (not in neural or segmental enhancer CSBs) were identified along with 104 additional *c*DTs enriched in mesodermal enhancers but also found at a lower frequency among neural and/or segmental enhancer CSBs. A common *c*DT-library was also generated that contains 993 *c*DTs that represent common sequence elements found in CSBs of both neural and mesodermal enhancers.

To search for enhancer sequence element conservation between taxa, we generated neural and mesodermal *c*DT-libraries from the combined alignments of mammalian and fly CSBs (Table 2) and many of the *c*DTs in these libraries align to both mammalian and fly CSBs. For example, the 11 bp neural specific *c*DT (CAGCTGACAGC) aligns with CSBs in the vertebrate Math-1 [27] and *Drosophila deadpan *[5] early CNS enhancers. All CSB-, *c*DT-libraries and alignment tools are available at the *cis-*Decoder website.

The constituent sequence elements of the different *c*DT-libraries are dependent on the enhancers used to identify them. As additional CSBs are included in the *c*DT-library construction, certain *c*DTs may be re-designated. For example, some that are currently considered neural specific will be discovered to be neural enriched, and others that are part of enriched libraries may be reassigned to common *c*DT-libraries.

Although each mammalian and fly *c*DT is present in at least two or more enhancers, most are not found as repeated sequences in any of the enhancers. In addition, one of the principle observations of our analysis is that enhancers of similarly regulated genes share different combinatorial sets of elements that are enhancer-type specific (see below).

Cross-library CSB alignments revealed that nearly all CSBs contain *c*DTs that are either shared by CSBs from divergent enhancer types or found only in CSBs from enhancers with related regulatory functions. For example, the 37 bp neural *mastermind *^#^10 CSB (TATTATTACTATATACAATATGGCATATTATTATTAC) contains a 9 bp sequence (first underlined sequence) also found in the 20 bp ^#^8 CSB from the *dpp *mesodermal enhancer [15,28] and it also contains a 14 bp sequence (second underlined sequence) that constitutes the entire 14 bp ^#^33 CSB from the neural enhancer region of *nerfin-1 *([29] and unpublished results).

The analysis of both the mammalian and fly common *c*DT-libraries reveals that many *c*DTs contain core recognition sequences for known transcription factors. However, when additional flanking CSB sequences are considered, many common transcription factor binding sites become tissue specific *c*DTs. For example, the DNA-binding site for basic helix-loop-helix (bHLH) transcription factors, the E-box motif CAGCTG (reviewed by [30]) is present 22 times in different neural CSBs, and 2 and 4 times within the CSBs of segmental and mesodermal enhancers, respectively. However, when flanking sequences are included in the analysis, such as the sequences CAGCTGG, CAGCTGAT, CAGCTGTG, CAGCTGCA, CAGCTGCT and ACAGCTGCC, all are neural specific *c*DTs (E-box underlined). It has been previously shown that different E-boxes bind different bHLH transcription factors to regulate different neural target genes [31]. Although transcription factor consensus DNA-binding sites are well represented in the *c*DT-libraries, greater than 50% of the *c*DTs in all of the libraries, both mammalian and fly, represent novel sequences whose function(s) are currently unknown. The fact that there exists such a high percentage of novel sequences within these highly conserved sequences indicates that the identity, function and/or the combinatorial events that regulate enhancer behavior are as yet unknown.

### *cis*-Decoder analysis of the murine Delta-like 1 enhancers identifies multiple shared elements with other related vertebrate embryonic enhancers

Although the resolution of *cis*-Decoder analysis increases as more enhancers and/or enhancer types are included in the CSB and *c*DT alignments, our analysis of mammalian enhancers found that many shared sequence elements can be identified among related enhancers when as few as two different enhancer groups are used to generate specific *c*DT-libraries. This is a particularly useful feature of *cis*-Decoder, especially when studying a biological process or developmental event where relatively little is known about the participating genes and their controlling enhancers. To demonstrate the ability of *cis*-Decoder to analyze relatively small subsets of enhancers, we show how *c*DT-libraries generated from 14 neural and 21 mesodermal mammalian enhancers can be used to distinguish between the neural and mesodermal enhancers that regulate embryonic expression of Dll1.

Dll1 encodes a Notch ligand that is essential for cell-cell signaling events that regulate multiple developmental events (reviewed by [32]). Studies in the mouse reveal that Dll1 is dynamically expressed in specific regions of the developing brain, spinal cord and also in a complex pattern within the embryonic mesoderm [33,34]. The 1.6 kb Dll1 *cis*-regulatory region, located 5' to its transcribed sequence, has been shown to contain distinct enhancers that direct gene expression in these different tissues [35]. These studies have identified two highly conserved neural enhancers, designated Homology I (H-I) and Homology II (H-II), and two mesodermal enhancers termed msd and msd-II. The H-I enhancer directs expression to the ventral neural tube, while the H-II enhancer primarily drives Dll1 expression in the marginal zone of the dorsal region of the neural tube [34]. The msd enhancer drives expression in paraxial mesoderm, and msd-II directs Dll1 expression to the presomitic and somitic mesoderm.

**Figure 2:**
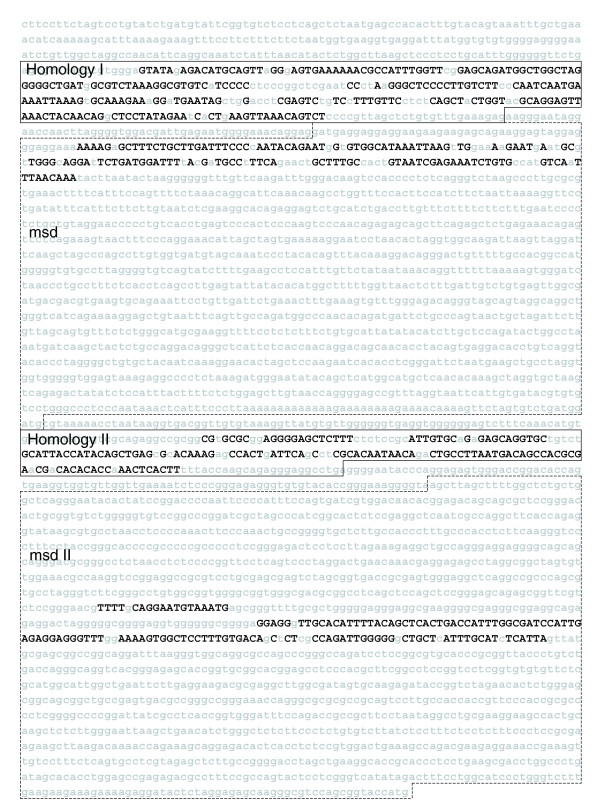
*EvoPrint *analysis of vertebrate Delta-like 1 enhancers. An *EvoPrint *of the vertebrate Dll1 *cis*-regulatory region generated from the following genomes: mouse (reference sequence), human, rhesus monkey, cow, rat, opossum and *Xenopus tropicalis*. Shown is the first codon (ATG) and 4,265 bp of upstream 5' flanking sequence of the mouse Dll1 gene containing, in 5' → 3' order, respectively, the Homology-I neural enhancer region (304 bp), the msd mesodermal enhancer (a 1,495 bp FokI restriction fragment), the Homology-II neural enhancer (207 bp fragment) and the msd-II mesodermal enhancer (1,615 bp *Hin*dIII restriction fragment) as described [35]. Multi-species conserved sequences within the murine DNA, shared by all orthologous DNAs that were used to generate the *EvoPrint*, are identified with uppercase black-colored letters and less or non-conserved DNA are denoted by lowercase gray-colored letters. Note that the chimpanzee, dog and chicken genomes were excluded from the analysis due either to sequence breaks and/or sequencing ambiguities as detected by *EvoDifference *profiles.

**Figure 3:**
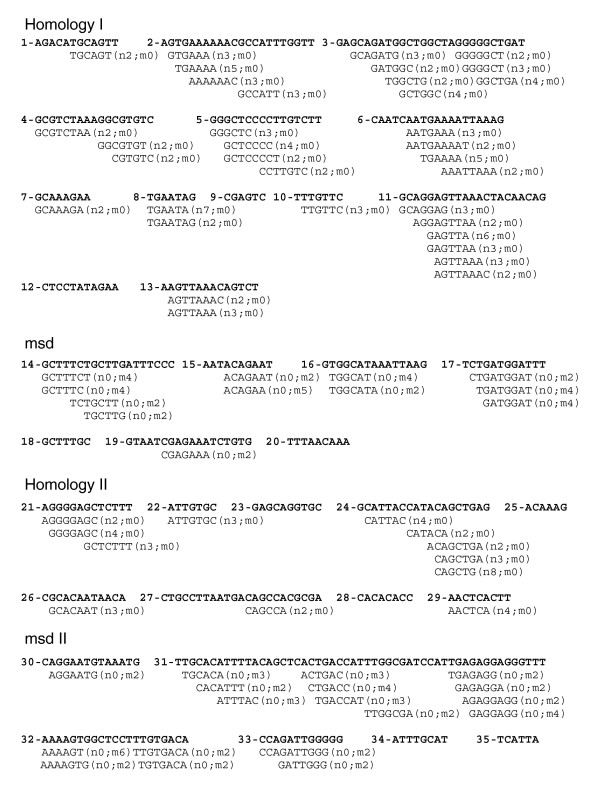
*cDT-scanner *analysis of vertebrate Delta-like 1 enhancers. Alignment of vertebrate neural and mesodermal specific *c*DTs with the Dll1 upstream CSBs identifies its neural and mesodermal enhancers. Dll1 CSBs of 6 bp or greater were curated using the *EvoPrint-parser *from the *EvoPrint *shown in Figure 2 and aligned with *c*DTs from the vertebrate neural and mesodermal *c*DT-libraries described in Table 2. Designations adjacent to the aligned *c*DTs indicate the number of perfect matches to CSBs within neural (n) or mesodermal (m) enhancers analyzed in this study. Transcription factor DNA-binding site searches of the Delta-like 1 CSBs and their aligning *c*DTs revealed that many contained putative binding sites and, in several cases, the shared sequence elements correspond exactly to, or had significant sequence overlap with, the characterized binding sites. For example, several *c*DTs that align to H-I enhancer CSBs correspond to known binding sites: these include a YY1 binding site (GCCATTT), an E-box (CAGATG; reviewed by [30]), a variant Oct1 site (ATGAAAAT) and a predicted core Lef-1 binding site (underlined) within a *c*DT (GCAAAGA). Within H-II conserved sequences, one common and one neural specific *c*DT aligned with the E-boxes (CAGGTG and CAGCTG), respectively.

An *EvoPrint *of the Dll1 *cis*-regulatory region reveals clustered CSBs in each of the enhancer regions (Figure 2). Here, *EvoPrint *analysis used mouse (reference DNA), human, rhesus monkey, cow, rat, opossum and *Xenopus tropicalis *orthologs, representing over approximately 240 My of collective evolutionary divergence. *EvoPrint-parser *CSB extraction of the *EvoPrint *generated a total of 35 CSBs of 6 bp or longer, representing 83% of the total MCS. A *c*DT-scan of the four Dll1 enhancer regions using the mammalian neural and mesodermal specific *c*DT-libraries accurately differentiates between the neural and mesodermal enhancers (Figure 3; note intra-CSB sequences are not shown). The *c*DT-library scan identified 77 type-specific sequence elements within the Dll1 CSBs and over half (52%) align with three or more CSBs from different enhancers, indicating that, even if Dll1 had been excluded from the analysis that generated the specific *c*DT-libraries, there would still be extensive coverage of the Dll1 CSBs by type-specific *c*DTs. All but eight of the CSBs contain elements that align with one or more neural or mesodermal specific *c*DTs. The H-I and H-II early CNS enhancers exhibited 64% and 43% coverage, respectively, by neural specific *c*DTs. The CSBs of the two mesodermal enhancers, msd and msd-II, exhibited 48% and 56% coverage, respectively, by one or more mesodermal specific *c*DTs. When common *c*DTs, shared by mesodermal and neural enhancers, were taken into account, coverage of all four enhancers was 81% (data not shown).

**Figure 4:**
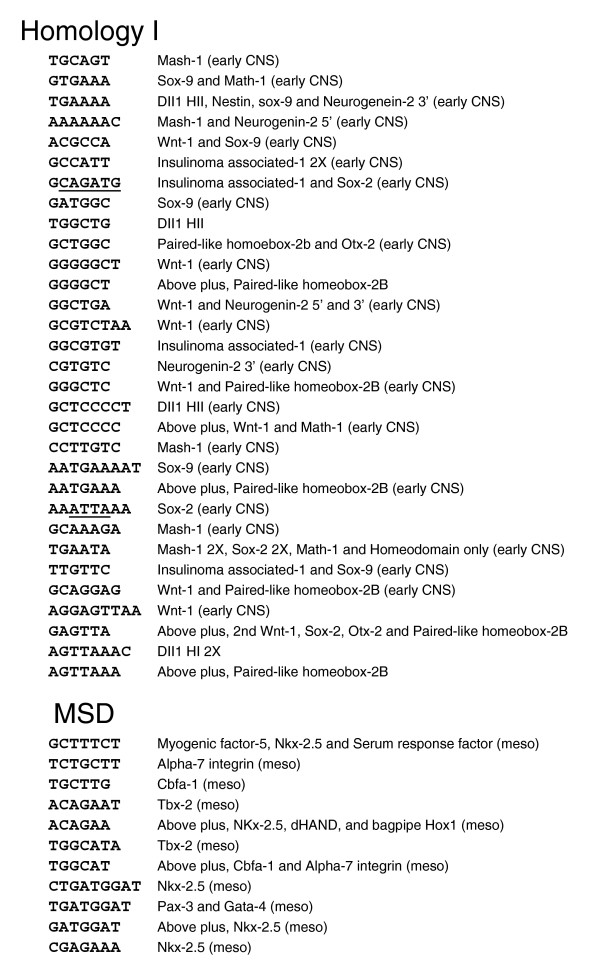
*cDT-cataloger *analysis of vertebrate *c*DTs that align with the Delta-like 1 Homology I and msd enhancers. *cDT-cataloger *analysis identifies other neural and mesodermal enhancers with shared sequence elements. Homeodomain protein DNA binding sites (ATTA) and bHLH binding sites known as E-boxes (CAGATG) are underlined. Analysis of Dll1 Homology II and msd2 enhancers is given in Additional data file 1.

*cDT-cataloger *analysis of aligning *c*DTs with H-I and H-II early CNS enhancers revealed that the H-I enhancer shares a remarkable 9 different sequence elements with the Wnt-1 early CNS neural plate enhancer CSBs [36], representing 62 bp (32%) of the H-I CSB coverage, 7 elements with the Paired-like homeobox-2b (Phox2b) hindbrain-sensory ganglia enhancer CSBs (23% coverage) and 6 sequence elements (20% coverage) with the Sox9^p ^hindbrain-spinal cord enhancer CSBs [37] as well as numerous other neural specific elements in common with CSBs of other neural enhancers (Figure 4; Additional data file 1). Comparisons of Dll1 H-I, Wnt-1, Phox2b and Sox9^p ^enhancer CSBs reveal that the orientation and order of the shared *c*DTs are unique for each of the enhancers (data not shown). The H-I and H-II enhancer CSBs also share the 7 bp sequence element GCTCCCC, and H-I has a repeat sequence element (AGTTAAA) that is present in two of its CSBs (^#^11 and ^#^13). The conserved AGTTAAA repeat is also part of a CSB in Phox2b enhancer [25]. *cDT-cataloger *analysis of the mesodermal enhancer *c*DT hits (Figure 4; Additional data file 1) reveal that, together, msd and msd-II share 7 elements in common with the mesodermal enhancer of Nkx2.5 [38] as well as numerous elements in common with CSBs of other mesodermal enhancers (Figure 2; Additional data file 1).

**Figure 5:**
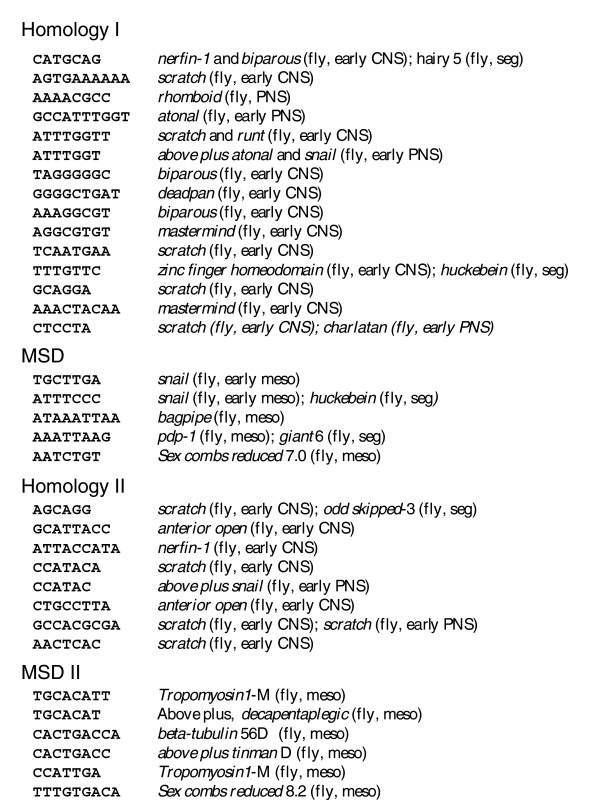
*cDT-cataloger *analysis of the Delta-like 1 upstream *c*DT hits using the combined mammalian and fly *c*DT-libraries. *cDT-cataloger *analysis using the combined mammalian and fly *c*DT-libraries (both neural and mesodermal specific libraries) identifies multiple Dll1 enhancer sequence elements (6 to 10 bp in length) that are shared among fly and mammalian enhancer CSBs. Note, only cDTs that align to *Drosophila *CSBs are shown.

Previous cross-taxa comparative studies have demonstrated that, in many cases, the regulatory circuits controlling the spatial-temporal regulatory activities of certain enhancers have been conserved over large evolutionary distances (discussed in [1]). For example, the *Deformed *autoregulatory element from *Drosophila *functions in a conserved manner in mice [39] and its human ortholog, the Hox4B regulatory element, provides specific expression in *Drosophila *[40]. Given this degree of conservation, we reasoned that *c*DT-libraries built from the combined alignments of enhancer CSBs from both mammalian and *Drosophila *CSB-libraries would lead to the discovery of additional enhancer type-specific sequence elements and thereby enhance our understanding of the relationship between evolutionarily distant enhancers (Table 2). By including all of the neural enhancer CSBs (286 mammalian and 601 *Drosophila*) in the CSB alignments, the total number of neural specific *c*DTs increased to 873 compared to 336 mammalian and 322 *Drosophila *neural specific *c*DTs (Table 2). The combined mesodermal specific *c*DT-library (Table 2) also increased compared to the individual mammalian and fly libraries. The combined mammalian and fly neural and mesodermal specific *c*DT-libraries contain *c*DTs that align with both mammalian and fly CSBs and *c*DTs that align exclusively with only mammalian or fly CSBs. Whether the 'cross-taxa' *c*DTs indicate significant functional overlap remains to be tested. However, a *c*DT-scan of the *EvoPrinted *Dll1 *cis-*regulatory region, using the cross-taxa libraries, identifies multiple conserved sequence elements that are shared with CSBs from functionally related fly enhancers (Figure 5), suggesting that many of the core *cis-*regulatory elements that participate in enhancer function are conserved across taxonomic divisions.

### *cis*-Decoder identifies sequence elements within the *Drosophila snail *and *hairy *stripe 1 enhancers that are also conserved in other functionally related tissue-specific enhancers

**Figure 6:**
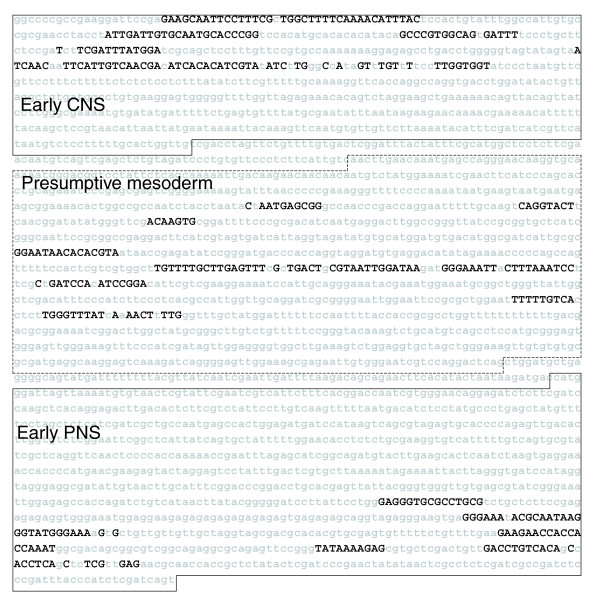
*EvoPrint *analysis of the *Drosophila snail cis*-regulatory region. An *EvoPrint *of the *Drosophila snail *upstream early CNS, presumptive mesodermal, and early PNS enhancer regulatory region (2,974 bp) [4,41] was generated using the following genomes: *D. melanogaster *(reference sequence), *D. sechellia*, *D. yakuba*, *D. erecta*, *D. ananassae*, *D. pseudoobscura*, *D. virilis*, *D. mojavensis *and *D. grimshawi*. Due to breaks in co-linearity, sequencing gaps and/or sequencing ambiguities, as detected by *EvoDifference *analysis, *D. simulans *and *D. persimilis *were not included in the analysis. Invariant MCSs, shared by all species, are identified with uppercase black-colored letters. The three previously identified genomic restriction fragments [4] containing the CNS, mesodermal and PNS enhancers are highlighted by solid lines for neural enhancers and dotted lines for the mesodermal enhancer.

To demonstrate the ability of *cis*-Decoder to differentiate between *Drosophila *neural and mesodermal enhancers, we show an analysis of the *snail *upstream *cis*-regulatory region. The enhancers that regulate *snail*'s dynamic embryonic expression have been mapped to a 2,974 bp upstream DNA fragment [4,41]. An *EvoPrint *of this sequence reveals that each of the restriction fragments that contain the different enhancer activities (CNS, mesodermal and PNS) harbor clusters of highly conserved CSBs (Figure 6). The combined evolutionary divergence of the *snail *upstream *EvoPrint *(generated from *Drosophila melanogaster*, *D. sechellia*, *D. yakuba*, *D. erecta*, *D. ananassae*, *D. pseudoobscura*, *D. mojavensis*, *D. virilis *and *D. grimshawi *orthologous sequences) is approximately 160 My, suggesting that many, if not all, of the identified CSBs are likely to be genus invariant and that each base-pair within a CSB has been evolutionarily challenged.

**Figure 7:**
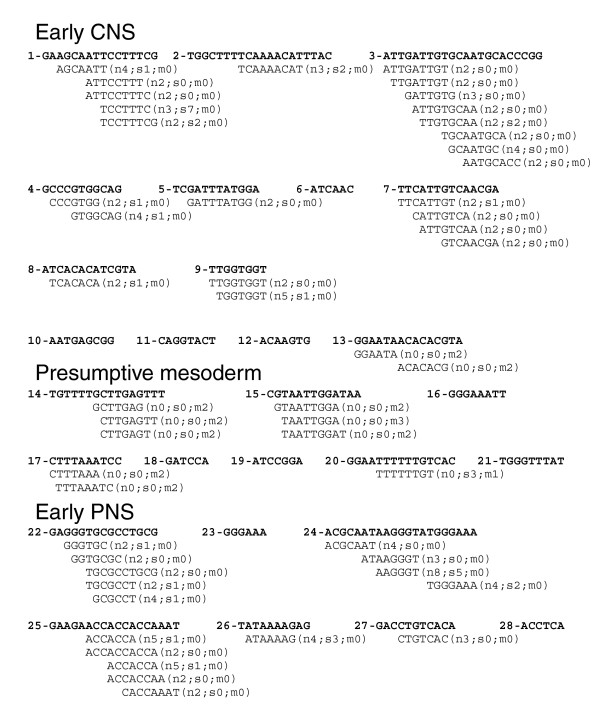
*cDT-Scanner *analysis of the *Drosophila snail *enhancer region. *c*DT-library scan of the *snail *enhancer region CSBs accurately differentiates between the neural, mesodermal and early PNS enhancers. Shown, in order of appearance within the *EvoP*, are 6 bp and greater CSBs aligned to *c*DTs from either the neural, segmentation or mesodermal *c*DT-libraries (described in Table 2). Designations adjacent to the aligned *c*DTs include number of perfect matches to neural (n), segmentation (s) and to mesodermal (m) enhancer CSBs analyzed in this study (enhancers used to generate *c*DT-libraries are listed in Table 1).

**Figure 8:**
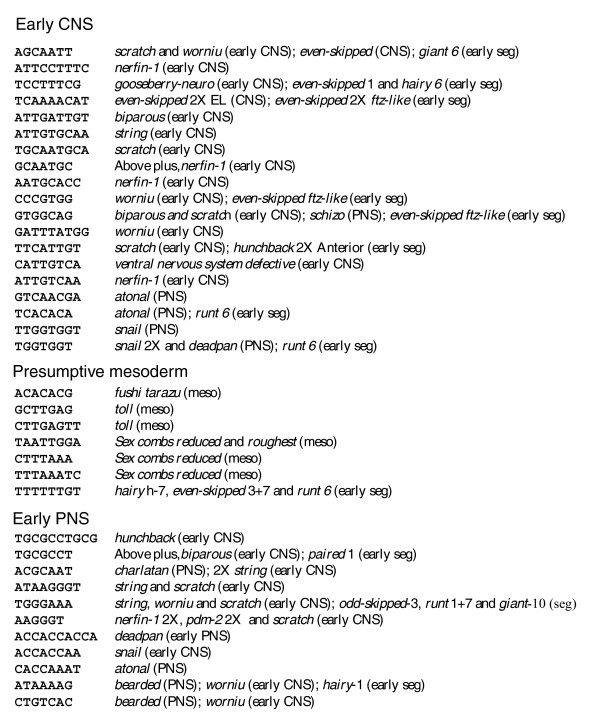
*cDT-cataloger *analysis of the *Drosophila snail *enhancers. *cDT-cataloger *analysis reveals that the different enhancers share sequence elements with the *snail *CNS, presumptive mesoderm, and PNS enhancers. Shown are *c*DTs identified in the *c*DT-scan (Figure 7) followed by the different enhancers that also contain the sequence in one or more of their CSBs (see Table 1 for enhancer references).

To identify sequence elements within the *snail *upstream CSBs that are present in CSBs of other functionally related or unrelated enhancers, we carried out a *c*DT-scan of the *snail EvoPrint *using the neural, segmental and mesodermal specific *c*DTs and the enriched *c*DT-libraries (Figure 7). Within the *snail *early CNS neuroblast enhancer region, our *c*DT-library scan identified 22 different neural and neural/segmental *c*DT hits, distributed among all but one of the CSBs, covering 73% of the CSBs. Interestingly, 10 of the 22 *c*DTs that align with the early CNS enhancer CSBs are found in CSBs of both neural and segmentation enhancers. The high percentage of neural/segmental *c*DT hits most likely reflects the fact that this enhancer initially drives *snail *expression in the neuroectoderm in a pair-rule pattern and then in a segmental pattern corresponding to the first wave of delaminating neuroblasts [4]. *cDT-cataloger *analysis of the aligning *c*DTs reveals that many of the identified sequence elements are also part of other early neuroblast enhancer CSBs. For example, the 9 bp *c*DTs ATTCCTTTC, ATTGATTGT, ATTGTGCAA, TGCAATGCA and GATTTATGG are also present, respectively, in CSBs from the *nerfin-1*, *biparous*, *string*, *scratch *and *worniu *neuroblast enhancers (Figure 8; see Table 1 for references).

Within the presumptive mesodermal enhancer CSBs, 11 *c*DTs mesodermal specific aligned with 5 of the 12 CSBs, covering 40% of the CSBs (Figure 7). Like the neural *c*DTs, some of the mesodermal *c*DTs contain putative DNA-binding sites for classes of known transcription factor families. For example, the seventh *c*DT (TAATTGGA) contains a consensus core DNA-binding sequence (underlined) for Antennapedia class homeodomain factors [42] (reviewed by [43]).

In the *snail *early PNS enhancer region, 5 of the 7 CSBs aligned with a total of 15 different *c*DTs that cover 69% of the total PNS CSB sequence (Figure 7). Similar to the CNS enhancer CSB *c*DT alignments, close to half of the PNS *c*DT hits represent sequence elements within both neural and segmental enhancer CSBs, again most likely a reflection of the segmental structure of the PNS. The significant overlap in *cDTs *found in both CNS and PNS enhancer CSBs may reflect the likelihood that many early neural specific transcriptional regulatory factors are pan-neural.

Many of the *snail *enhancer CSB-*c*DT hits represent sequences found only in two CSBs, *snail *itself and one other. In these instances it appears that these elements, although specific for neural or mesodermal CSBs, are relatively rare when compared to others. Only through analysis of additional enhancers will it be clear whether these rare elements are indeed type-specific or only enriched in the type-specific CSBs. Nevertheless, the fact that the sequence elements identified by these rare *c*DTs are conserved in two distinct enhancer CSBs that have both been under positive selection for over 160 My of collective divergence merits their inclusion in the analysis.

As part of our study of *Drosophila *enhancers, we carried out *cis*-Decoder analysis of 38 segmentation enhancers responsible for both gap and pair-rule gene expression during *Drosophila *embryogenesis. Although the segmentation enhancer specific library consisted of only 284 *c*DTs, these *c*DTs aligned with over 70% of bases of the CSBs of segmentation enhancers. As an example of alignment of these cDTs with a segmental enhancer, we present an alignment of segmentation specific cDTs with the *hairy *stripe 1 enhancer (Additional data file 2). *cis*-Decoder recognizes highly conserved Abdominal-B, HOX, Hunchback, Kruppel and Tramtrack binding sites, as well as additional uncharacterized sites, as being shared by *hairy *stripe 1 enhancer and other segmentation enhancers.

### *Full-enhancer scanner *identifies less conserved repeated *c*DTs and CSBs

Previous studies have demonstrated that certain enhancers, particularly those controlling the dynamic expression of developmental genes, contain clusters of DNA-binding site motifs for specific transcription factors (for example, see [44,45]; reviewed by [46]). Comparative genomic studies of orthologous enhancers have also revealed that, within a binding site cluster, individual DNA-binding sites can undergo turnover (discussed in [47,48]). This loss of and/or gain of transcription factor docking sites during evolution suggests that the repeated motifs may be functionally redundant and that the stability of any one binding site is most likely due to selective pressure(s) to maintain: total number of binding sites for tight spatial/temporal regulation; functional interactions between a bound factor and adjacent factors and/or; competition between antagonistic regulatory factors for overlapping binding sites. For example, overlapping/linked binding sites have been identified in the 3' most CSB of the *Krüppel *central domain enhancer [9,10]. The 15 bp CSB (CTGAACTAAATCCG) contains overlapping sites for the transcriptional activator Bicoid and repressor Knirps proteins [11]. *In vivo *experiments reveal that these interlocking sites are functionally important [12]. Additional binding sites for both of these factors are also present in the *Krüppel *enhancer but not all are found in CSBs (data not shown).

**Figure 9:**
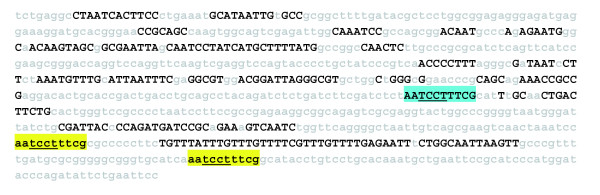
*Full enhancer scanner *analysis identifies less conserved sequences that are also part of conserved sequence blocks. The following *Drosophila *species were used to produce an *EvoPrint of *the *Drosophila melanogaster *800 bp *even-skipped *stripe ^#^1 enhancer [18]: *D. melanogaster *(reference sequence), *D. simulans*, *D. sechellia*, *D. erecta*, *D. ananassae*, *D. persimilis*, *D. pseudoobscura*, *D. virilis*, *D. mojavensis *and *D. grimshawi*. *Drosophila yakuba *was not included in the *EvoPrint *analysis due to lack of sequence co-linearity detected with *EvoDifference *prints. Invariant MCSs, shared by all species used to generate the *EvoPrint*, are identified with uppercase black-colored letters. A *Full-enhancer scan *of the enhancer with one of its 10 bp CSBs (blue highlight) revealed that it is repeated two additional times in the less conserved inter-block sequences (lowercase yellow highlighted sequences). Note that the underlined sequence in this CSB is the core DNA-binding sequence for the Tramtrack transcription factor.

The *Full-enhancer scanner *is used to identify less conserved repeated *c*DTs by rescanning the entire enhancer sequence with the aligning *c*DTs. For example, a *Full-enhancer scan *of the *even-skipped *stripe 1 enhancer with its aligning *c*DTs reveals that the ^#^15 CSB (AATCCTTTCG) is present two additional times within the intra-CSB sequences (Figure 9). Interestingly, this CSB contains the consensus binding sequence for Tramtrack (underlined), a regulator of segmental gene expression [49]. *EvoDifference *analysis reveals that the 5' most inter-block (AATCCTTTCG) is conserved in all *Drosophila *species except *D. ananassae *and the 3' inter-block repeat is absent in six of the ten species used to generate the *EvoPrint *(data not shown).

### Use of *cis*-Decoder to examine novel *cis*-regulatory sequences

**Figure 10:**
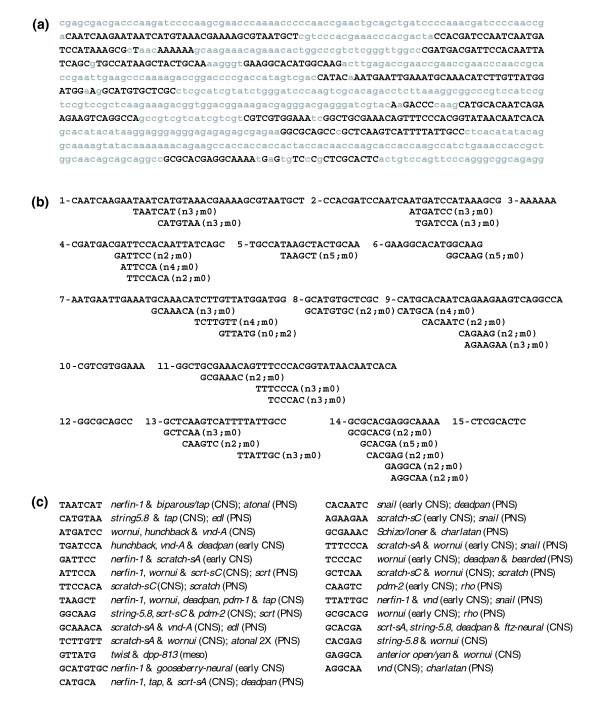
*cis*-Decoder analysis of the *Drosophila HLHmβ *5' upstream *cis*-regulatory region. *cis*-Decoder analysis of the *Drosophila HLHmβ *upstream region identifies neural enhancer sequences. **(a) **An *EvoPrint *of the 869 bp *Drosophila HLHmβ cis*-regulatory region [54] was generated using the following genomes: *D. melanogaster *(reference sequence), *D. simulans*, *D. sechellia*, *D. yakuba*, *D. erecta*, *D. ananassae*, *D. persimilis*, *D. pseudoobscura*, *D. virilis*, *D. mojavensis *and *D. grimshawi*. Uppercase nucleotide sequences are conserved in all of the above genomes. **(b) ***cis*-Decoder tag analysis of the *HLHmβ *enhancer CSBs. CSBs (6 bp or greater) were extracted from the *EvoPrint *shown in (a) and aligned with *Drosophila c*DTs from neural and mesodermal libraries. Designations adjacent to the aligned *c*DTs include number of perfect matches to neural (n) and mesodermal (m) enhancers analyzed in this study. **(c) ***cDT-cataloger *analysis of the aligning *c*DTs reveal that the *HLHmβ *enhancer contains elements shared with 26 other neural enhancer CSBs and one mesodermal CSB.

One major use of the *cis*-Decoder methodology is the comparative analysis of different enhancer regions. To test *cis*-Decoder's efficacy in characterizing putative *cis*-regulatory regions that were not included in the preparation of the *c*DT-libraries, we have examined a number of genes both in *Drosophila *and vertebrates using *EvoPrinter *and *c*DT-library scans. Our analysis reveals that putative enhancer regions associated with CNS-expressed genes align with a higher proportion of neural-specific *c*DTs than with mesodermal-specific *c*DTs. For example, *cis*-Decoder analysis of the immediate upstream regions from *Drosophila E(spl) region transcript mβ *(*HLHmβ*) [50] and of the human gene encoding Tuberoinfundibular peptide of 39 residues (TIP39) [51-53] revealed that both of these neural expressed genes had significant coverage by neural-specific *c*DTs of their proximal cis-regulatory region CSBs. Figure 10 shows *cis*-Decoder analysis of *HLHmβ*, while our analysis of TIP39 is presented in Additional data file 3.

During embryonic development, *HLHmβ *expression is activated in the ventral neurogenic ectoderm immediately prior to neuroblast delamination [50,54] and enhancer-reporter constructs from the *HLHmβ *enhancer region [55] are expressed in proneural territories in the ventral ectoderm at the time of the first wave of neuroblast delamination(stages 9-10) and in neuroblasts (Figure 1 of [55]). Our *EvoPrint *analysis of the 883 bp enhancer region (Figure 10a) revealed that 338 bases were highly conserved, and over 90% of these were found in CSBs of 6 or more bases. Alignment of *Drosophila *neural-specific and mesodermal-specific *c*DTs revealed that 11 of the 15 *HLHmβ *CSBs aligned with a total of 28 neural specific *c*DTs, while only 1 of its CSBs aligned with a single mesodermal specific *c*DT (Figure 10b,c). Both proneural transcription factors and the Notch pathway, acting through the Su(H) transcription factor, are implicated in the regulation of E(spl) complex genes (reviewed by [56]). Among the *c*DTs aligning with the CSBs, one, GCATGTGC, contains an E-box (underlined), the focus of activity of proneural transcription factors, and two others, TTTCCCA and TCCCAC, align with the consensus Su(H) binding site.

Although higher specificity is obtained by alignment with *c*DTs of 7 bases or greater, we have found that it is not unusual for 80% of CSBs associated with neural expressed genes to align with neural-specific *c*DTs versus only 20% of the CSBs in the same putative enhancer regions aligning with mesodermal-specific *c*DTs even when 6 base long *c*DTs are included in the analysis (data not shown). As the size and specificity of these libraries grow, their use as predictors of enhancer function will most likely increase as well.

As an additional assessment of the specificity of *c*DT-library scans, we generated negative control CSB-libraries for alignment to *c*DTs. These datasets, both *Drosophila *and mammalian, consisted of conserved sequence blocks within exons of genes that are not predominantly expressed in the CNS (data not shown). For this analysis we use the percent coverage of CSBs by *c*DTs, as used above for the analysis of Dll1 enhancers in which we counted the percent of the bases in the CSBs that aligned with cDTs. Whereas *Drosophila *and mammalian neural-specific *c*DTs, including hexamers, cover approximately 56% and 70%, respectively, of CSBs from neural enhancers, alignment with control CSBs was 20% or less. Again, when the alignment was repeated with cDTs of 7 bp or greater the CSB coverage of neural sequence was 5-fold greater than that observed with the control datasets. Taken together, our *c*DT alignments demonstrate their utility in identifying enhancer type-specific conserved sequence elements.

Evaluation of the *cis*-Decoder method was also carried out by examining the contribution that each enhancer made to the *c*DT-libraries. As one adds new enhancer CSBs to a specific library, the number of *c*DTs increases, such that alignment coverage of enhancer type-specific CSBs also increases. We illustrate the contribution of each enhancer to the specific *cDT-libr*aries in our study (Additional data file 4). Overall, for *Drosophila *enhancers, prior to their inclusion in a library, on average 41% of the conserved nucleotides of enhancers align with the tissue specific cDT-library appropriate for that enhancer, while after inclusion in a library, 65% of the conserved nucleotides align. For example, addition of the *bearded *proneural enhancer [57], consisting of 21 CSBs (a total of 303 bp), to the *Drosophila *neural-specific CSB library resulted in 26 new neural-specific *c*DTs that were shared with at least one other neural enhancer. Prior to its inclusion, coverage of the *bearded *CSBs by alignment of neural-specific *c*DTs was 43%, while after its inclusion in the *c*DT-library preparation the alignment coverage of its CSBs increased to 67%. Addition of new enhancers to the out-group, used to remove common *c*DTs from a specific library, also enhances the specificity of the type-specific library and frequently shifts *c*DTs from specific to enriched libraries. Taken together, increased specificity of an enhancer-type *c*DT-library can be achieved either by including new similarly regulated enhancers in the generation of the *c*DT-library or increasing the number of out-group CSBs used to remove non-specific *c*DTs. Ideally, both approaches should be pursued to increase the depth and resolution of a particular *c*DT-library.

## Conclusion

This study describes a systematic approach for the identification and comparative analysis of highly conserved DNA sequences within enhancers. Because our approach focuses solely on conserved sequences, the probability that *cis*-Decoder analysis dissects functionally important DNA is greatly enhanced. Most of the 2,086 CSBs identified in this study have undergone negative selection during more than 160 My of collective evolutionary divergence. Alignment of hundreds of CSBs from both similarly regulating enhancers and functionally different enhancers assures that conserved *cis-*regulatory elements shared by as few as two enhancers are identified and included in the analyses. Our *c*DT-scans show that most CSBs have a modular organization made up of smaller overlapping/interlocking sequence elements that align with CSBs of other enhancers. A typical CSB is made up of both enhancer type-specific sequence elements and common elements that are found in enhancers with different regulatory functions and, surprisingly, more than half of all of the shared CSB sequence elements do not correspond to know transcription factor DNA-binding sites and, as of yet, are functionally novel.

*c*DT-library scans of *EvoPrinted cis-*regulatory DNA reveal that it is possible to differentiate between functionally different enhancer types before any experimental/expression data are known. For example, *c*DT-library scans of the mammalian Dll1 or *Drosophila snail cis-*regulatory DNA sequences accurately differentiate between neural and mesodermal enhancers (Figures 3 and 7). *c*DT-library scans of co-regulating enhancers, using multiple libraries, reveal the combinatorial complexity of the *cis-*regulatory sequence elements involved in coordinate gene expression. Our studies indicate that many co-regulating enhancers rely on different combinations of the tissue-specific *cis-*regulatory elements to achieve synchronous regulatory behaviors. Although not highlighted in this paper, information gleaned from the *c*DT-scans and subsequent *cDT-cataloger *analysis of multiple co-regulating enhancers can be used to construct 'higher resolution' *c*DT-libraries that harbor many, or most, of the sequence elements that direct coordinate gene expression.

For example, sub-libraries of the *Drosophila *neural specific library can be generated to identify neuroblast- and PNS-specific tags. Enhancer CSB analysis using *c*DT-libraries generated from the combined alignments of both mammalian and fly CSBs also suggests that many of the sequence elements represented by the different *c*DTs have been conserved across taxonomic divisions and may represent core elements used by many metazoans to direct tissue-specific gene expression patterns.

Although we have initially generated *c*DT-libraries from general classes of different enhancer types, this approach should be applicable to the analysis of gene co-regulation in any cell type involved in any biological event. As the variety and depth of the different *c*DT-libraries increase, we believe that *c*DT-library scans of *EvoPrinted *putative enhancer regions will have great utility for the identification and initial characterization of *cis*-regulatory sequences. Future efforts that address the role of individual enhancer CSBs and the dissection of their modular elements will undoubtedly yield new insights into the function of these 'evolutionarily hardened' sequences and ultimately produce a better understanding of the regulatory code underlying coordinate gene expression.

## Materials and methods

*cis*-Decoder [26] is a six-step integrated series of protocols and web-based algorithms that can be used to identify evolutionarily conserved DNA sequences that are shared among different enhancers (Figure 1). The following sections provide a detailed description of each step of the *cis*-Decoder procedure: *EvoPrint *analysis [58], for the discovery of MCSs; *EvoPrint-parser*, for CSB extraction and annotation; *CSB-aligner*, for the identification of shared elements between CSBs; *cDT-scanner*, to reveal *c*DT positions and their relations to other *c*DTs within CSBs; *Full-enhancer scanner*, for the discovery of less-conserved repeated *c*DTs or CSBs within enhancers; and *cDT-cataloger *for the identification of enhancers with shared sequence elements. A more detailed description of these steps is given at the *cis*-Decoder website. The Java applets *CSB-aligner*, *cDT-scanner*, *Full-enhancer scanner *and *cDT-cataloger *are available on-line at the *cis*-Decoder website and can be downloaded to the users computer to avoid Java-web browser incompatibilities. In our experience, a current version of the Mozilla browser avoids many potential incompatibilities.

### *EvoPrinter*

The first step in the *cis*-Decoder analysis of an enhancer is preparing CSB-libraries from enhancers with related and/or divergent expression patterns. Enhancer CSBs were identified by the phylogenetic footprinting algorithm *EvoPrinter *[9]. Unlike other multi-species alignment programs that identify CSBs by outputting multiple aligned sequences interrupted by sequence gaps to optimize alignments, *EvoPrinter *outputs a single uninterrupted sequence to reveal CSBs as they exist in a species of interest. In *Drosophila*, when 9 or more species are used to generate an *EvoPrint*, the combined mutagenic histories of all of the orthologous DNAs represent an excess of 160 My of collective evolutionary divergence, thus affording near base-pair resolution of the functionally important DNA within the species of interest (discussed in [9]). Likewise, *EvoPrint *analysis of orthologous DNAs that include placental mammals (human, chimpanzee, rhesus monkey, cow, dog, rat and mouse), and, optionally, the opossum, detects CSBs that have been maintained for over 200 My of collective divergence. The *EvoPrinter *and *EvoDifference *print analysis algorithms and companion protocols are described [9], and are found online at the *EvoPrinter *tutorial website.

### *EvoPrint-parser*

The *EvoPrint-parser *is a JavaScript program that automatically extracts and generates reverse-complement sequence and then annotates and lists in their 5' to 3' order CSBs that are 6 bp or longer from a known or putative enhancer region. Tissue-specific enhancer CSB-libraries can then be generated by assembling CSBs from enhancers of known function (for example, neural or mesodermal enhancers).

### *CSB-aligner*

*CSB-aligner *is a Java applet that allows one to identify short sequence elements shared between different CSBs. To generate a CSB-alignment, parsed CSBs from multiple enhancer regions are placed in the upper window of the *CSB-aligner *applet. Then, forward direction CSBs from one or more enhancers are placed in the lower window of the CSB-aligner. A box associated with the lower window of the *CSB-aligner *allows for the naming of the CSBs introduced into the lower box and selection of the minimum aligned length (6, 7 or 8 base windows have been routinely used). Output length of the alignments produced by *CSB-aligner *can be selected (default value 100 bases).

Output of the *CSB-aligner *consists of the CSBs that were input into the lower window aligned with the CSBs that were introduced into the upper window. The *CSB-aligner *does not record CSB self-alignments. A second output window, the results table, is a list of the aligned matches along with their positions. Each of the output columns of the results table can be sorted by selecting the column header of the column to be sorted. Contents of results tables can be copy-pasted into Microsoft Word.

The CSB-alignment can be saved as an HTML file. Saving the HTML file allows copy pasting from the saved file into Microsoft Word and, once in Word, the file can be reformatted and saved or printed as the original readout. The *CSB-alignment *program has functioned successfully with the introduction of thousands of CSBs in both windows. The following CSB-libraries were created from *EvoPrints *of enhancers listed in Table 1: mammalian neural, mammalian mesodermal, *Drosophila *neural, *Drosophila *mesodermal and *Drosophila *segmental.

### Interpreting the *CSB-aligner *readout and generation of *c*DT-libraries

A *c*DT is a short sequence element of 6 bp or greater that is a perfect match to sequences within CSBs that are present in two or more enhancers. A *c*DT-library represents a collection of *c*DTs that are shared by the various enhancers examined. Two types of *c*DT-libraries have been generated in this study. First, a 'tissue-specific library' contains *c*DTs that are shared by a group of enhancers that regulate similar expression patterns but are absent from a second set of enhancers that direct expression in tissues outside of the first group. Second, a 'common *c*DT-library' contains *c*DTs that were shared between sets of enhancers of divergently regulated genes. A subset of common libraries included 'enriched' libraries that had a three-fold greater representation from one enhancer type (for example, neural) than from a second type (for example, mesodermal).

All libraries were generated from readouts of the *CSB-aligner*. Making enhancer-type specific libraries requires two different CSB-libraries generated from functionally different enhancers, a library from the tissue of interest (for example, neural), and a second library that serves as an 'out-group' (for example, mesodermal). For the generation of a neural *c*DT-library, neural CSBs in both forward and reverse directions were copy-pasted into both upper and lower windows of *CSB-aligner*. The resulting *c*DTs from this alignment are listed in the 'Result of CSB alignment table' of the *CSB-aligner *output, in the column titled 'Motif.' Since this *c*DT list contains multiple copies of different *c*DTs, the extra copies are removed using the Java applet Puzzamatic 1.0 [59], a freeware created by Ron Surratt. The *c*DT list that contains all unique cDTs is then alphabetized and sorted by size also using Puzzamatic 1.0. The cDTs, constituting a raw neural *c*DT-library, were then copy/pasted into a Microsoft Word document. A second CSB-alignment is then performed with the neural CSBs in the top window of *CSB-aligner*, and mesodermal CSBs in the lower window. The *c*DTs from this alignment were freed of extra copies as above. These *c*DTs constituted an unedited common neural/mesodermal *c*DT-library. The unedited neural and common *c*DT-libraries are combined and *c*DTs common to the two libraries (present in the first and second alignments) are removed using the JavaScript program *cDT-cleaner *[60], thus leaving only the neural-specific sequences. Neural enriched and common *c*DTs were curated from the unedited shared *c*DT-library.

For *Drosophila*, segmental, neural (treating CNS and PNS specific enhancers together), and mesodermal specific *c*DT-libraries were generated. The out-group for neural and segmental *c*DT-libraries was the mesodermal CSB-library, and the out-group for the mesodermal *c*DT-library was neural CSBs. For mammals, neural and mesodermal *c*DT-libraries were generated. All *c*DT-libraries are listed in Table 2 and full libraries are available online [26].

### Identification of shared elements within enhancers with the *c*DT-*scanner*

The function of *cDT-scanner *is to determine the relationship between any enhancer and any other group of MCSs used to generate the CSB libraries. *cDT-scanner *aligns the *cDTs *contained within various *c*DT-libraries with CSBs within an *EvoPrint*. *cDT-scanner *is a Java applet that uses a variant of the *cis*-Decoder aligner; it looks for only perfect matches between *c*DTs and CSB sequences. Alignment of *c*DTs using *cDT-scanner *is accomplished by first pasting a *c*DT-library in the upper window of *cDT-scanner *and then pasting the *EvoPrint *or CSBs to which they are to be aligned in the lower window. The output of *cDT-scanner *consists of perfect matches of *c*DTs aligned under the input CSBs. Since each library consists of *c*DTs shared by different enhancers, *cDT-scanner *portrays the shared elements within each CSB. A *cDT-scanner *alignment should be saved; information from saved files can be copy-pasted into Microsoft Word without loss of formatting features. For details on how to format *c*DT-alignments, see the website. A second output window for the *cDT-scanner*, a results table, is a list of the aligned matches along with their positions. Selecting the output column header sorts the results under that header. Contents of results tables can then be copy-pasted into Microsoft Word.

### Finding less-conserved sequence elements

The '*Full-enhancer scanner*' is a Java applet that identifies additional repeated *c*DT or CSB sequences within less conserved sequences flanking CSBs of enhancers. For this alignment, *c*DTs or CSBs present within an enhancer can be curated from the output of *cDT-scanner *termed 'Results from *c*DT-scan.' Curate both forward and reverse/complement sequences and paste into the upper window of *Full-enhancer scanner*. The *EvoPrinted *enhancer should be copy-pasted into the lower window. The program aligns to both conserved and non-conserved sequences of the *EvoPrint*.

### Identification of enhancers that share conserved elements using *cDT-cataloger*

*cDT-cataloger *uses a variant of the *CSB-aligner*; it records only perfect matches between CSBs and *c*DTs of a specified size. The output lists those CSBs containing perfect sequence matches to the *c*DTs, and can be used to identify enhancers and count the number of times each *c*DT aligns with any CSB-library. Cataloguing is accomplished by copy-pasting the CSB-libraries (both forward and reverse directions) into the upper window of the *cDT-cataloger *and the selected *c*DTs of a single uniform size in the lower window. The size of the *c*DT(s) must be entered into the window provided.

## Additional data files

The following additional data are available with the online version of this paper. Additional data file 1 contains the *cDT-cataloger *analysis of the murine Delta-like 1 Homology-II and msd-II enhancers supplemental to Figure 4. Additional data file 2 contains the *cis*-Decoder analysis of the *Drosophila hairy *stripe 1 enhancer. Additional data file 3 is a figure that contains *cis*-Decoder analysis of the human TIP39 5' proximal promoter. Additional data file 4 is a table that documents the contribution of each *Drosophila *and mammalian enhancer to the specific *c*DT-libraries generated in this study.

## Supplementary Material

Additional data file 1 *cDT-cataloger *analysis of the murine Delta-like 1 Homology-II and msd-II enhancers supplemental to Figure 4 Click here for file

Additional data file 2 *cis*-Decoder analysis of the *Drosophila hairy *stripe 1 enhancer Click here for file

Additional data file 3 *cis*-Decoder analysis of the human TIP39 5' proximal promoter Click here for file

Additional data file 4 Contribution of each *Drosophila *and mammalian enhancer to the specific *c*DT-libraries generated in this study Click here for file
