# Supplementary material for: cis-Decoder discovers constellations of conserved DNA sequences shared among tissue-specific enhancers
Source: Genome Biol. 2007 May 9;8(5):R75. doi: 10.1186/gb-2007-8-5-r75 (PMC1929141; doi:10.1186/gb-2007-8-5-r75)
Supplement: Additional data file 1 — cDT-cataloger analysis of the murine Delta-like 1 Homology-II and msd-II enhancers supplemental to Figure 4 [file gb-2007-8-5-r75-S1.doc]

***c*DT-cataloger analysis of the murine Delta-like 1 Homology-II and msd-II enhancers**

**AGGGGAGC** Delta-1 HI (early CNS)

**GGGGAGC** above plus, Wnt-1 and Math-1 (early CNS)

**GCTCTTT** Paired-like homeobox-2B (early CNS)

**ATTGTGC** Delta-1 HII 2X and Sox-9 (early CNS)

**CATTAC** Math-1, Paired-like homeobox-2B and Mash-1 (early CNS)

**CATACA** Sox-9 (early CNS)

**ACAGCTGA** Sox-9 (early CNS)

**CAGCTGA** above plus, Math-1 (early CNS)

**CAGCTG** above plus, Otx-2 (early CNS)

**GCACAAT** Delta-1 HII 2X and Sox-9 (early CNS)

**CAGCCA** Delta-1 HI (early CNS)

**AACTCA** Otx-2, Wnt-1, Sox-2 and Paired-like homeobox-2B (early CNS)

**AGGAATG** Bagpipe homeobox gene-1 (meso)

**TGCACA** Stem cell leukemia factor 2X (meso)

**CACATTT** Myogenic factor-5 (meso)

**ATTTAC** Tbx-2 (meso)

**ACTGAC** Six-2 2X and HoxA-5 (meso)

**CTGACC** Six-2, Alpha-7 Integrin and Tbx-1 (meso)

**TGACCAT**  Stem cell leukemia factor and Hairy/enhancer of split-7 (meso)

**TTGGCGA** Nkx-2.5 (meso)

**TGAGAGG** Hairy/enhancer of split-7 (meso)

**GAGAGGA** Stem cell leukemia factor (meso)

**AGAGGAGG** Alpha-7 Integrin (meso)

**GAGGAGG** above plus, Gata-6 (meso)

**AAAAGTG** Nkx-2.5 (Meso)

**AAAAGT** above plus, Mef-2C (meso)

**CCAGATTGGG**Tbx-1 (meso)

**GATTGGG** above plus, 2^nd^ Tbx-1 (meso)

*c*DT-cataloger analysis identifies enhancers with shared sequence elements. Hox sites (ATTA) and E-boxes (CANNTG) are underlined.
