# Supplementary material for: cis-Decoder discovers constellations of conserved DNA sequences shared among tissue-specific enhancers
Source: Genome Biol. 2007 May 9;8(5):R75. doi: 10.1186/gb-2007-8-5-r75 (PMC1929141; doi:10.1186/gb-2007-8-5-r75)
Supplement: Additional data file 2 — cis-Decoder analysis of the Drosophila hairy stripe 1 enhancer [file gb-2007-8-5-r75-S2.doc]

***cis*-Decoder analysis of the *Drosophila hairy* stripe 1 enhancer**

(a)

**taacttggtccagtggcgtgtgagctacgtctaaccctatgtatatagatatagatatagatatagatagatatagatagatagacagggatagaggtatacctgcacccaccacgccCATAATCCTTTTATGGCAAAtAAACAAACAacGTCACGGcgactcagtcgcgaaggattgccatggcTAGGTAAAAAgtgccaAAAGGGTGCAATAAAccgggttgagatggccaaagggtatggccacggtatggcatggttatagatatggaaggatagggaagagttgttgcACACGCGcggtggcaTTGTTGCACactCgGTTTTATGGGTGTGTGGTTTTAGCCCTTTGATGgGCATGATCATCCTGTTAATAGAAAgcgtggaaacccgttgcaacttgtaaccctacctgcacgcccaaccaatctcTAATCCTTTCcgcggcgatcctgAACCCTTTtcggtccagctcgtttggagggggcGAGCGTGTCtAAggcGATTTAATTAACGCGGTcGcAACTAAgccaccgaataccaggacatggcttaaatcggaaccctttcgctcggccaggaggaggtggccctgctaaaagCCatccgGCTACTGTTCCgaggacgggttaaggatgcatggatgcagtagctacgtgggcgtcatcggaaaagtcagacagggagcgggtgggcggaaggatttgacttccagcgaaaggattgccggacttaactttcggaaaaactccaATATTTACCAAAtaccaaagggccaccaagtgcactttgttcagcacttggccagcagtcgaaaagtcaagtctggttttgggatttcaacagcaaattaaccggctgagccggcaaagcgag**

(b)

**1-CATAATCCTTTTATGGCAAA 2-AAACAAACA 3-GTCACGG 4-TAGGTAAAAA**

---ATAATCCTTT(n0;s2;m0) TAGGTAAA(n0;s2;m0)

----TAATCCT(n0;s8;m0) TAGGTAA(n0;s3;m0)

-----AATCCT(n1;s16;m0) AGGTAAA(n4;s4;m0)

-----AATCCTTT(n0;s12;m0) AGGTAAAAA(n1;s2;m0)

------ATCCTTT(n3;s14;m0) GGTAAAA(n3;s3;m0)

--------CCTTTTA(n2;s2;m0)

---------CTTTTATG(n1;s2;m0)

----------TTTTATGGC(n0;s2;m0)

----------TTTTATGG(n1;s7;m0)

--------------ATGGCAAA(n1;s2;m0)

**5-AAAGGGTGCAATAAA 6-ACACGCG 7-TTGTTGCAC 8-GTTTTATGGGTGTGTGGTTTTAGCCCTTTGATG**

AAAGGGT(n1;s2;m0) TTTTATGG(n1;s7;m0)

---AAGGGT(n8;s5;m0) TTTATGGG(n0;s2;m0)

----AGGGTG(n2;s1;m0) TGGGTGT(n2;s2;m0)

---- GGGTGC(n2;s1;m0) GGTGTGT(n1;s2;m0)

-------GTGCAAT(n2;s2;m0) TGTGGTTT(n1;s2;m0)

--------TGCAATA(n2;s4;m0) TGTGGTT(n1;s3;m0)

--------TGCAATAAA(n1;s3;m0) TGGTTTTA(n0;s2;m0)

---------GCAATAAA(n2;s4;m0) GTTTTAGC(n0;s2;m0)

TTTTAGCC(n0;s2;m0)

AGCCCTT(n0;s2;m0)

**9-GCATGATCATCCTGTTAATAGAAA 10-TAATCCTTTC 11-AACCCTTT**

--GCATGAT(n0;s2;m0) TAATCCT(n0;s8;m0) AACCCTT(n2;s3;m0)

----ATGATCA(n0;s3;m0) AATCCT(n1;s16;m0) AACCCT(n2;s5;m0)

-----TGATCAT(n0;s3;m0) AATCCTTT(n0;s12;m0) ACCCTTT(n2;s2;m0)

-----TGATCA(n0;s8;m0) ATCCTTT(n3;s14;m0) ACCCTT(n8;s5;m0)

------GATCATCC(n2;s2;m0)

------GATCATC(n3;s3;m0)

-------ATCATCC(n3;s2;m0)

--------------TGTTAATA(n1;s2;m0)

TAATAGA(n0;s2;m0)

ATAGAAA(n2;s1;m0)

**--------------------------------------------------------------------------------12-GAGCGTGTC 13-GATTTAATTAACGCGGT 14-AACTAA 15-GCTACTGTTCC 16-ATATTTACCAAA**

---GAGCGT(n4;s2;m0)ATTTAATT(n3;s2;m0) GCTACTG(n0;s2;m0)ATATTTAC(n3;s2;m0)

----AGCGTG(n5;s2;m0)TTTAATTA(n3;s3;m0) CTACTG(n0;s4;m0) ATTTACC(n3;s3;m0)

----------------------TTAATTAA(n0;s2;m0) CTGTTC(n0;s2;m0)------ ----------- CGCGGT(n0;s2;m0)

**ATAATCCTTT** *paired* (early seg)

**TAATCCT** above plus, *ftz* CE8, *giant*-10, *hairy*-1, *hairy*-6, *odd-skipped*-3, *paired* and *runt*-7

**AATCCTTT** *eve*-1 and -5, *ftz* 2X CE8,, *runt*-7, *prd* E, *odd-skipped*-3 and *giant* -10 (early seg)

**ATCCTTT** above plus, *paired* 2X (early seg)

**TTTTATGGC** *eve*-4 (early seg)

**TTTTATGG** above plus, *eve*-7, *giant*-3, *hairy*-1 2X and *runt*-5 (early seg); *nerfin-1* (early CNS)

**ATGGCAAA** *pdm-1* central domain (early seg)

**TAGGTAAA** *odd-skipped*-5 (early seg)

**TAGGTAA** above plus, *runt*-3 (early seg)

**AGGTAAAAA** *eve*-5 (early seg); *charlatan* (PNS)

**AGGTAAA** above plus, *odd-skipped*-3 (early seg); *vnd* and *deadpan* (early CNS)

**GGTAAAA** *eve*-5, *hairy*-1 and -h7 (early seg); *nerfin-1*, *pdm-2* (early CNS); *rho* and *char* (PNS)

**AAAGGGT** *nerfin-1* (early CNS)

**GTGCAAT** *giant*-3 (early seg); *nerfin-1* and *snail* (early CNS)

**TGCAATAAA** *giant*-3 and *eve*-2 (early seg); *vnd* (early CNS)

**TGCAATA** above plus, *biparous* (early CNS)

**GCAATAAA** *caudal*-14, *eve*-2 and *giant*-3 (early seg); *string* (early CNS)

(c)

**TTTATGGG** *giant*-10 (early seg)

**TGGGTGT** *paired*-0 (early seg); *eyeless* (CNS)

**TGTGGTT** *runt* 2X -7 (early seg); *gooseberry-neuro* (early CNS)

**GTTTTAGC** *runt*-6 (early seg)

**TTTTAGCC** *eve*-5 (early seg)

**AGCCCTT** *paired*-2 (early seg)

**GCATGAT** *eve*-1 (early seg)

**ATGATCA** *paired*-1 (early seg)

**GATCATCC** *hunchback* Anterior (early seg); *hunchback* and *worniu* (early CNS)

**GATCATC** above plus, *eve*-1 (early seg)

**TGTTAATA** *runt*-3+7 (early seg); *bearded* (PNS)

**TAATAGA** *caudal*-14 (early seg)

**GAGCGT** *odd-skipped*-3 (early seg); *scratch* 2X (early CNS)

**TTTAATTA** *hairy*-3+4 and *odd-skipped*-5 (early seg); *nerfin-1*, *worniu* and *string* (early CNS)

**CGCGGT** *giant*-3 (early seg)

**GCTACTG** *caudal*-14 (early seg)

**CTACTG** above plus, *paired* cc 2X (early seg)

**ATATTTAC** *paired* O-E (early seg); *hunchback* and *runt* (early CNS); *bearded* (PNS)

**ATTTACC** *giant*-10 and *odd-skipped*-5 (early seg); *runt* and *vnd* (early CNS)

*c*DT-library scans of the *Drosophila hairy* stripe #1 enhancer *EvoPrint* identify repeated sequence elements within CSBs and elements shared within CSBs from other enhancers. **(a)** An *EvoPrint* of the 876 bp *Drosophila hairy* stripe 1 *cis*-regulatory region [17] was generated using the following genomes: *D. melanogaster* (reference sequence), *D. simulans*, *D. sechellia*, *D. yakuba*, *D. erecta*, *D. ananassae*, *D. persimilis*, *D. pseudoobscura*, *D. virilis*, *D. mojavensis* and *D. grimshawi*. Uppercase nucleotide sequences are conserved in all of the above genomes. Blue-colored highlighted sequences denote a conserved repeat that contains adjacent core DNA-binding motifs for homeodomain (TAAT) and Tramtrack (TCCT) transcription factors. Underlined repeat sequences represent core DNA-binding sequences for the Hunchback Zn-finger transcription factor. Hairy (green) and Krüppel (yellow) DNA-binding sites are also denoted. **(b)** *cis*-Decoder tag analysis of the *hairy* stripe 1 enhancer CSBs. CSBs (6 bp or greater) were extracted from the EvoPrint shown in panel A and aligned with *Drosophila* *cDTs* from neural, mesodermal and segmental libraries. Designations adjacent to the aligned *c*DT include number of perfect matches to neural (n), segmentation (s) and to mesodermal (m) enhancers analyzed in this study. **(c)** *cDT-catalogs* of the aligning *c*DTs reveal that the *hairy* stripe 1 enhancer contains repeated sequence elements (underlined) and elements shared with other segmental and neural enhancer CSBs but not with mesodermal specific *c*DTs.

**Background**

During early embryonic development, the segmentation gene *hairy* is expressed in a striped pair-rule expression pattern in the cellular blastoderm [119]. The enhancer that controls *hairy* expression in the first anterior stripe, the stripe 1 enhancer, has been delimited to a 876 bp sequence located ~ 5 kb 5’ to the transcribed sequence [17]. An *EvoPrint* of this region, generated from 10 species (see below), identifies a cluster of CSBs and a *c*DT-scan of the *EvoPrint*, using the fly neural, segmental and mesodermal *c*DT-libraries, reveals that 11 of the 16 CSBs aligned with a total of 20 segmentation specific and 37 segmentation enriched/neural *c*DTs (see below); none of the CSBs aligned with mesodermal or neural specific *c*DTs. Over 77% of the aligning *c*DTs represent sequences found in three or more CSBs. A *c*DT-scan using the common *c*DT-library revealed that the 5 CSBs that did not contain segment specific *c*DT alignments all aligned with common *c*DTs (data not shown). The total *c*DT coverage of the stripe 1 enhancer CSBs is 65% for the segmental *c*DTs and nearly 100% when the common *c*DT coverage is included; this was similar to the coverage of other segmentation CSBs (data not shown).

Transcription factor DNA-binding site searches of the CSBs and the aligning *c*DTs revealed that some, but not all, of the CSB sequences coincide with consensus transcription factor DNA-binding sites. For example, the core consensus DNA-binding sites for Tramtrack (TCCT, [49]), Krüppel (AACCCTT, [24]), Hunchback/Castor (TTTTATG: [84,120,121]), Abdominal B (TTTATGG, [122]) and Hairy (CACGCG, [123]) transcription factors are present in the *hairy* stripe 1 CSBs, suggesting that some or all of these factors may play a role in the regulation of this enhancer in the cellular blastoderm or may function by silencing it later in development. Except for the Hairy core recognition sequence, which is found among the common *c*DTs, the core binding sequences for Tramtrack, Krüppel, Hunchback/Castor and Abdominal B are part of larger *c*DTs that are present in segmental and neural CSBs (see below). Although the binding site searches identified multiple factors that may participate in stripe 1 enhancer regulation, more than 70% of the conserved sequences were not covered by known transcription factor docking sites and the majority of these novel sequences correspond to *c*DT identified elements that are conserved in multiple related or divergently regulating enhancers.
