# Supplementary material for: cis-Decoder discovers constellations of conserved DNA sequences shared among tissue-specific enhancers
Source: Genome Biol. 2007 May 9;8(5):R75. doi: 10.1186/gb-2007-8-5-r75 (PMC1929141; doi:10.1186/gb-2007-8-5-r75)
Supplement: Additional data file 3 — cis-Decoder analysis of the human TIP39 5' proximal promoter [file gb-2007-8-5-r75-S3.doc]

***cis*-Decoder analysis of the human TIP39 gene 5’ proximal promoter**

(a)

**tcctttctttttctgtcgttcccattctttctctctatttcttcctcggccactctgtctttgtccttctctttgtgtctctgttcctctgac****ggtccttccgccaGtCTCTATTTTTAgCCCTCTGACACACCcCcTGTgtccacctctcTGTCTGTctgtctctccccctccctcgtctcaggtccagcttctGGTCCCAATTAGTtGGTGgcgGCCAaGGCAGcgGCaGGtcCCCCACccccGGCTcCTCATTACCGCTGGCgGCTCCTAATGAGCcTGGggagggggtgaccccgcgtccccggccccccggcCTGCGTCACTGcCcggtGCGGGGGCTGcggaggcgatataagggggctgccaccatcgctgccccagcccactgcacg**

**1-CTCTATTTTTA 2-CCCTCTGACACACC 3-TGTCTGT 4-GGTCCCAATTAGT 5-CCCCAC**

(b)

**CTCTAT(n3;m0) CTCTGACA(n0;m2)**

**TCTATT(n3;m0)**

**6-CTCATTACCGCTGGC 7-GCTCCTAATGAGC 8-CTGCGTCACTG 9-GCGGGGGCTG**

**CTCATT(n3;m0) CCTAAT(n2;m0) GGGGGCT(n2;m0)**

**CATTAC(n4;m0) AATGAG(n3;m0)**

**GCTGGC(n4;m0) ATGAGC(n2;m0)**

**CTCTAT** Math-1, Neurogenin-2 3’ and Sox-2 (CNS)

(c)

**TCTATT** Math-1, Phox-2b and Sox-2 (CNS)

**CTCTGACA** dHAND and Gata-4 (meso)

**CTCATT** Math-1 2X and Otx-2 (CNS)

**CATTAC** Math-1, Phox-2b, Mash-1 and Delta-1 HI (CNS)

**GCTGGC** Phox-2b 2X, Delta-1 HI and Otx-2 (CNS)

**CCTAAT** Sox-9p and Wnt-1 (CNS)

**AATGAG** Math-1 2X and Otx-2 (CNS)

**ATGAGC** Math-1 and Sox-2 (CNS)

**GGGGGCT** Wnt-1 and Delta-1 HI (CNS)

*c*DT-library scans of a TIP39 proximal promoter *EvoPrint* identify sequence elements within CSBs that are also found in vertebrate neural enhancers. **(a)** An *EvoPrint* of the 395 bp TIP39 proximal promoter region was generated using the following genomes: human (reference sequence), rhesus, dog, cow mouse and rat. Uppercase nucleotide sequences are conserved in all of the above genomes. **(b)** *cis*-Decoder *c*DT-scanner analysis of the TIP39 proximal promoter region. CSBs (6 bp or greater) were extracted from the *EvoPrint* shown in panel A and aligned with vertebrate *c*DTs from neural and mesodermal libraries. Designations adjacent to the aligned *c*DT include number of perfect matches to neural (n) and to mesodermal (m) enhancers analyzed in this study. **(c)** *cDT-catalogs* of the aligning *c*DTs reveal that the TIP39 proximal promoter region contains 9 *c*DTs shared with neural enhancers and one *c*DT (underlined) that is present in two mesodermal enhancers.
