# Supplementary material for: cis-Decoder discovers constellations of conserved DNA sequences shared among tissue-specific enhancers
Source: Genome Biol. 2007 May 9;8(5):R75. doi: 10.1186/gb-2007-8-5-r75 (PMC1929141; doi:10.1186/gb-2007-8-5-r75)
Supplement: Additional data file 4 — Contribution of each Drosophila and mammalian enhancer to the specific cDT-libraries generated in this study [file gb-2007-8-5-r75-S4.doc]

**Individual enhancer contribution to the generation *c*DT-libraries**

These tables show the percent *c*DT coverage of CSBs of each enhancer before and after its inclusion in the *c*DT-library preparation. Evaluation of the *cis*-Decoder method was carried out by examining the contribution that each enhancer made to the *c*DT-libraries. This study also evaluated the ability of *cis*-Decoder to discover prospective enhancer regions that were not included in the initial analysis. CSBs of each enhancer in the study were individually removed from the analysis to discover *c*DTs and then new *c*DT-libraries were generated.  The extent to which each new library covered by alignment the CSBs of the removed enhancer was determined and compared to the coverage by the full *c*DT-library.

***Drosophila* enhancers**

| **Enhancer** | **Total bp of extracted CSBs** | ***c*DT coverage of CSBs**  **prior to their inclusion** | **% *c*DT coverage of CSBs prior to their inclusion** | ***c*DT coverage of CSBs**  **after their inclusion** | **% *c*DT coverage of CSBs after their inclusion** |
| --- | --- | --- | --- | --- | --- |
| *anterior open/yan* | 179 | 67 | 37 | 117 | 65 |
| *atonal* F:2.6 PNS | 310 | 166 | 54 | 259 | 84 |
| *bagpipe* DS3.5 | 483 | 56 | 12 | 163 | 34 |
| *β-tub*56DAS | 101 | 21 | 21 | 52 | 51 |
| *bearded* PNS | 303 | 130 | 43 | 203 | 67 |
| *caudal* +14 construct | 116 | 68 | 59 | 101 | 87 |
| CG15151/*PFE* | 122 | 64 | 52 | 73 | 60 |
| *charlatan* | 331 | 151 | 46 | 257 | 78 |
| *dpn* CNS | 114 | 109 | 96 | 63 | 55 |
| *dpp* 813 | 192 | 65 | 34 | 86 | 45 |
| *edl* PNS | 153 | 65 | 42 | 101 | 66 |
| *eve* neuronal CNS | 134 | 46 | 34 | 83 | 62 |
| *eve ftz*-like | 283 | 115 | 41 | 220 | 78 |
| *eve* stripe 3+7 | 80 | 50 | 63 | 61 | 76 |
| *eve* stripe1 | 230 | 120 | 52 | 157 | 68 |
| *eve* stripe2 | 77 | 30 | 39 | 47 | 61 |
| *eve* stripe4 6 | 156 | 100 | 64 | 122 | 78 |
| *eve* stripe5 | 135 | 73 | 54 | 98 | 73 |
| *eve* EL CNS | 186 | 39 | 21 | 80 | 43 |
| *eve* MES | 43 | 0 | 00 | 16 | 37 |
| *eyeless* 12 PNS | 259 | 145 | 56 | 165 | 64 |
| *ftz* PS4 | 49 | 29 | 59 | 38 | 78 |
| *ftz* CE8024 | 320 | 109 | 34 | 176 | 55 |
| *ftz* distal | 131 | 8 | 06 | 38 | 29 |
| *ftz* neuro CNS | 190 | 44 | 23 | 71 | 37 |
| *gooseberry*-fragIV | 103 | 22 | 21 | 62 | 60 |
| *gooseberry-n* CNS | 135 | 49 | 36 | 100 | 74 |
| *gooseberry* GLE | 87 | 51 | 59 | 72 | 83 |
| *giant* 1 | 42 | 27 | 64 | 29 | 69 |
| *giant* 10 | 195 | 137 | 70 | 177 | 91 |
| *giant* 3 | 145 | 98 | 68 | 115 | 79 |
| *giant* 6 | 170 | 33 | 19 | 95 | 56 |
| *hairy* h7 | 155 | 61 | 39 | 105 | 68 |
| *hairy* stripe 6+2 | 107 | 61 | 57 | 89 | 83 |
| *hairy* stripe 0 | 60 | 35 | 58 | 40 | 67 |
| *hairy* stripe 1 | 206 | 116 | 56 | 150 | 73 |
| *hairy* stripe 3+4 | 180 | 86 | 48 | 125 | 69 |
| *hairy* stripe 5 | 159 | 126 | 79 | 137 | 86 |
| *hunchback* CNS | 127 | 62 | 49 | 104 | 82 |
| *hunchback* anterior | 64 | 34 | 53 | 38 | 59 |
| *hunchback* upstream | 267 | 113 | 42 | 192 | 72 |
| *huckebein* ventral | 159 | 32 | 20 | 101 | 64 |
| *heartless* early | 60 | 14 | 23 | 43 | 72 |
| *knirps* 5 | 187 | 77 | 41 | 121 | 65 |
| *Kruppel* CD1 | 193 | 156 | 81 | 178 | 92 |
| *mastermind* | 177 | 97 | 55 | 139 | 79 |
| *Mef2* I-D | 68 | 29 | 43 | 57 | 84 |
| *Mef2* II-E | 86 | 24 | 28 | 42 | 49 |
| *nerfin-1* CNS | 667 | 393 | 59 | 373 | 56 |
| *odd skipped*-3 | 312 | 165 | 53 | 210 | 67 |
| *odd skipped*-5 | 146 | 79 | 54 | 122 | 84 |
| *pdm-1* Gap+CNS | 116 | 56 | 48 | 90 | 78 |
| *pdm-2* CE8012 | 109 | 68 | 62 | 91 | 83 |
| *pdp1* intron 1 | 298 | 47 | 16 | 114 | 38 |
| *pdp1* intron 2 | 185 | 36 | 19 | 84 | 45 |
| *paired* cc | 140 | 49 | 35 | 78 | 56 |
| *paired* O-E | 212 | 85 | 40 | 153 | 72 |
| *paired* stripe P | 63 | 24 | 38 | 48 | 76 |
| *paired* stripe 1 | 85 | 27 | 32 | 61 | 72 |
| *paired* stripe 2P | 119 | 25 | 21 | 77 | 65 |
| *paired* zebra | 169 | 48 | 28 | 94 | 56 |
| *rhomboid* | 265 | 141 | 53 | 197 | 74 |
| *runt* 6 | 564 | 172 | 30 | 355 | 63 |
| *runt* 7 | 248 | 108 | 44 | 173 | 70 |
| *runt* stripe 5 | 144 | 64 | 44 | 98 | 68 |
| *runt* stripe 3+7 | 204 | 66 | 32 | 159 | 78 |
| *runt* 15G CNS | 106 | 35 | 33 | 77 | 73 |
| *Schizo/loner* PNS | 174 | 79 | 45 | 133 | 76 |
| *Scr* 3.0RR | 320 | 71 | 22 | 150 | 47 |
| *Scr* 7.0RR | 766 | 122 | 16 | 301 | 39 |
| *Scr* 8.2XX | 1415 | 206 | 15 | 616 | 44 |
| *scratch* PNS | 234 | 114 | 49 | 161 | 69 |
| *scratch* sA | 701 | 259 | 37 | 465 | 66 |
| *scute* | 97 | 42 | 43 | 63 | 65 |
| *semaphorin* 1a | 227 | 104 | 46 | 162 | 71 |
| *snail* CNS | 116 | 61 | 53 | 96 | 83 |
| *snail* PNS | 90 | 58 | 64 | 71 | 79 |
| *snail* MES | 101 | 7 | 07 | 48 | 48 |
| *serpent*-A7.1EB | 105 | 0 | 00 | 55 | 52 |
| *string* b-5.8 CNS | 234 | 101 | 43 | 143 | 61 |
| *target of PoxN* | 638 | 414 | 65 | 458 | 72 |
| *tinman* B | 64 | 0 | 00 | 14 | 22 |
| *tinman* C | 141 | 15 | 11 | 65 | 46 |
| *tinman* D | 61 | 6 | 10 | 41 | 67 |
| *Tkr* | 130 | 68 | 52 | 90 | 69 |
| *toll*-6.5RL | 461 | 82 | 18 | 251 | 54 |
| *Tropomysin1*-M | 30 | 13 | 43 | 25 | 83 |
| *Tropomysin1*-P | 79 | 29 | 37 | 52 | 66 |
| *teashirt*-del-1-5 | 56 | 17 | 30 | 28 | 50 |
| *twist*-del | 135 | 28 | 21 | 79 | 59 |
| *vnd* | 75 | 46 | 61 | 48 | 64 |
| *vnd* A | 75 | 49 | 65 | 56 | 75 |
| *worniu* | 932 | 367 | 39 | 642 | 69 |
| *zfh2* | 93 | 26 | 28 | 65 | 70 |
| **AVERAGE** | **208** | **80** | **41** | **130** | **65** |

| **Enhancer** | **Total bp of extracted CSBs** | ***c*DT coverage of CSBs**  **prior to their inclusion** | **% *c*DT coverage of CSBs prior to their inclusion** | ***c*DT coverage of CSBs**  **after their inclusion** | **% *c*DT coverage of CSBs after their inclusion** |
| --- | --- | --- | --- | --- | --- |
| α7 integrin | 127 | 56 | 44 | 72 | 57 |
| Bagpipe Hox1 | 140 | 43 | 31 | 87 | 62 |
| Cbf1 non-coding | 296 | 103 | 35 | 162 | 55 |
| Dll1 H1 CNS | 186 | 88 | 47 | 140 | 75 |
| Dll1 H2 CNS | 107 | 47 | 44 | 56 | 52 |
| Dll1 msd | 91 | 29 | 35 | 46 | 55 |
| Dll1 msdII | 103 | 40 | 39 | 71 | 69 |
| Forkhead box 1 | 162 | 50 | 31 | 85 | 52 |
| Gata4 | 198 | 80 | 40 | 112 | 57 |
| Gata6 | 296 | 97 | 33 | 185 | 63 |
| dHAND | 98 | 45 | 46 | 75 | 77 |
| Hes 7 | 109 | 31 | 28 | 67 | 61 |
| Homeodomain only | 94 | 13 | 14 | 41 | 44 |
| HoxA-5 | 149 | 26 | 17 | 62 | 42 |
| IA-1 CNS | 310 | 90 | 29 | 149 | 48 |
| Mash1 CNS | 391 | 93 | 24 | 262 | 67 |
| Math1 CNX | 490 | 191 | 39 | 346 | 71 |
| Mef2c | 213 | 61 | 29 | 109 | 51 |
| Myogenic factor-5 | 319 | 105 | 33 | 161 | 50 |
| Nestin CNS | 118 | 64 | 54 | 90 | 76 |
| Nfatc1 | 39 | 21 | 54 | 23 | 59 |
| Neurogenin 2:3' | 240 | 87 | 36 | 161 | 67 |
| Neurogenin 2:5’ | 353 | 138 | 39 | 267 | 76 |
| Nkx-2.5 | 341 | 113 | 33 | 197 | 58 |
| Otx 2 CNS | 327 | 130 | 40 | 228 | 70 |
| Pax 3 | 108 | 50 | 46 | 62 | 57 |
| Phox2B CNS | 327 | 165 | 50 | 260 | 80 |
| Stem cell leukemia | 100 | 21 | 21 | 29 | 29 |
| Six2 | 154 | 92 | 60 | 124 | 81 |
| Sox-2 CNS | 312 | 96 | 31 | 165 | 53 |
| Sox-2 #2 CNS | 82 | 40 | 49 | 57 | 70 |
| sox9^p^ | 291 | 118 | 41 | 198 | 68 |
| Serum response f | 173 | 63 | 36 | 119 | 69 |
| Tbx1 | 390 | 136 | 35 | 227 | 58 |
| Tbx2 | 228 | 133 | 58 | 202 | 89 |
| Wnt-1 | 438 | 180 | 41 | 291 | 66 |
| **AVERAGE** | **219** | **104** | **41** | **172** | **66** |

**Mammalian enhancers**
